# Functional Heterogeneity of the Young and Old Duplicate Genes in Tung Tree (*Vernicia fordii*)

**DOI:** 10.3389/fpls.2022.902649

**Published:** 2022-06-20

**Authors:** Lan Jiang, Tingting Fan, Xiaoxu Li, Jun Xu

**Affiliations:** ^1^Key Laboratory of Non-coding RNA Transformation Research of Anhui Higher Education Institution, Yijishan Hospital of Wannan Medical College, Wuhu, China; ^2^Central Laboratory, Yijishan Hospital of Wannan Medical College, Wuhu, China; ^3^Clinical Research Center for Critical Respiratory Medicine of Anhui Province, Wuhu, China; ^4^The Laboratory of Forestry Genetics, Central South University of Forestry and Technology, Changsha, China; ^5^Technology Center, China Tobacco Hunan Industrial Co., Ltd., Changsha, China; ^6^Hunan Institute of Microbiology, Changsha, China

**Keywords:** young and old duplicate gene, *Vernicia fordii*, evolution, asymmetric function, evolutionary properties

## Abstract

Genes are subject to birth and death during the long evolutionary period. Here, young and old duplicate genes were identified in *Vernicia fordii*. We performed integrative analyses, including expression pattern, gene complexity, evolution, and functional divergence between young and old duplicate genes. Compared with young genes, old genes have higher values of Ka and Ks, lower Ka/Ks values, and lower average intrinsic structural disorder (ISD) values. Gene ontology and RNA-seq suggested that most young and old duplicate genes contained asymmetric functions. Only old duplicate genes are likely to participate in response to *Fusarium* wilt infection and exhibit divergent expression patterns. Our data suggest that young genes differ from older genes not only by evolutionary properties but also by their function and structure. These results highlighted the characteristics and diversification of the young and old genes in *V. fordii* and provided a systematic analysis of these genes in the *V. fordii* genome.

## Introduction

Like all organisms, genes also undergo birth and death during the long evolutionary period. Following a gene duplication event, two identical copies of the ancestral gene perform exactly the same function, which may result in the death of some genes (Ota and Nei, 1994; Nei et al., 1997). According to the order of genes generation, they can be classified as old genes and young genes. Young genes retention may be closely related to the accommodation of environmental changes compared to old genes (Kaessmann, 2010; Song et al., 2019). Gene retroposition, horizontal transfer, duplication, recombination, or originating from non-genic sequences may be the source of these genes (Long et al., 2003, 2013; Kaessmann, 2010). So far, many researchers have studied the relationship between expression patterns and evolutionary patterns of young and old genes. Compared to old genes, young genes have a higher intrinsic structural disorder (ISD), shorter gene length, lower histone modification, lower gene expression level, more relaxed purifying selection, and faster evolutionary rate (Wolf et al., 2009; Capra et al., 2010; Vishnoi et al., 2010; Cui et al., 2015; Wang et al., 2016; Banerjee and Chakraborty, 2017; Wilson et al., 2017; Song et al., 2019). Old genes usually play an important role in the growth and development of organisms. In contrast, most young genes may not be necessary for plant growth and development, but a few of them may acquire new essential functions in the survival of new environments (Chen et al., 2010, 2012). Young genes tend to evolve more quickly and experience weaker purifying selection than old genes. Additionally, for young genes, duplicates experience weaker stronger translational selection than singletons and old genes (Yin et al., 2016).

So far, although there are many studies on the characteristics of young and old genes, both of function and characteristics of young and old duplicate genes produced by gene duplication events are still excluded. Compared with animals, plants have experienced one or more whole-genome duplication (WGD) events (Schranz et al., 2012). In addition, some plants have also undergone small-scale duplication (SSD) events along with their growth and development (Conant and Wolfe, 2008; Cao et al., 2019c). In general, duplicates that are retained by SSD are mainly involved in cell death, stress response, and metabolism, while duplicates formed by WGD participate in signal transduction, kinases, and development (Maere et al., 2005; Pareek et al., 2006; Corrochano et al., 2016). Although the genes produced by WGD and SSD may differ in their function, gene duplication events will produce new copies (i.e., young genes) that cause subfunctionalization or neofunctionalization to promote adaptive evolution and increase relative fitness in plants (Gottlieb, 1982; Flagel and Wendel, 2009; Van De Peer et al., 2017).

*Vernicia fordii*, as a unique industrial oil tree species in China, is a monecious plant with wide distribution and many varieties (Cao et al., 2019b). There are great differences in the yield of tung oil among different *V. fordii* varieties. Young and old duplicate genes may plant a significant role in this process. Previous researchers have sequenced the whole genome and RNA-seq of *V. fordii* (Chen et al., 2016; Cui et al., 2018; Liu et al., 2019), so we analyzed the characteristics of young and old duplicating genes in *V. fordii*. According to the synonymous substitution ratio (Ks) value for *V. fordii*, the young and old duplicate gene pairs were classified in the present study. We also compared gene complexity, gene expression patterns, and evolutionary patterns between young and old duplicate genes in *V. fordii*. This study may help us to further understand the functional divergence and evolution of duplicate genes in *V. fordii*.

## Materials and Methods

### Identification of Young and Old Duplicate Genes in *Vernicia fordii*

To identify the duplicate genes in *V. fordii*, we used the strict evaluation criteria as follows: (1) *E*-value ≤ 10^–10^, (2) identity >80%, and (3) length of aligned sequences >80% of the length of each sequence, as described by Clevenger et al. (2016) and Song et al. (2019). The young and old duplicate genes from *V. fordii* using a method described in Song et al. (2019). Briefly, the top and bottom 25% of Ks values for gene pairs were defined as old and young duplicate gene pairs, respectively (Song et al., 2019).

### Chromosomal Location, Gene Ontology, Sequence Complex, and Substitution Rates

The chromosomal location of *V. fordii* genes was obtained from the *V. fordii* genomic annotation file. According to the sequencing name, the chromosomal location of each young and old duplicate gene was determined in *V. fordii* genome. The gene ontology for each young and old duplicate gene pair was generated using Blast2GO software against the NR database (Conesa et al., 2005). Ka/Ks (non-synonymous to synonymous substitution ratio), Ka, and Ks were determined using the aligned CDS in the Codeml procedure PAML software (version 4.4) all alignment gaps were deleted (Yang, 2007). Polypeptide length and Fop (frequency of optimal codons), for the young and old duplicate gene pairs were calculated using CodonW software (version 1.4.2).^1^ GC1 (GC content at the first codon site), GC2 (GC content at the second codon site), and GC3 (GC content at the third codon site) were estimated using an in-house Perl script. The IUPred2A online tool was used to estimate ISD with default parameters (Mészáros et al., 2018).

### RNA-Seq Data

The raw sequences for 17 different tissues (PRJNA483508 and PRJNA445068) were filtered using the cutadapt software (version 1.8.1) (Martin, 2011). The high-quality reads were mapped to the *V. fordii* genome using HISAT2 software (version 2.1.0) with default parameters (Pertea et al., 2016). The StringTie software (version 2.0) was used to obtain FPKM (fragments per kilobase of exon model per million reads mapped) values for all young and old duplicate genes (Pertea et al., 2015, 2016). Gene-expression breadth is a measure of the number of tissues where a gene matched at least one tissue, and this value was also calculated in *V. fordii* (Jordan et al., 2005; Cao et al., 2019a).

The raw sequences for *V. fordii* root tissue infected by *Fusarium* wilt into three periods, including 2 dpi (i.e., the early stage), 8 dpi (i.e., the subsequent stage), and 13 dpi (i.e., the final stage), each with three biological replicates, obtained from NCBI Gene Expression Omnibus with accession number GSE80228 (Chen et al., 2016). The DESeq package was used to determine the differentially expressed transcription factors (DETs) with a fold-change ≥ 2 and *p*-value ≤ 0.05 (Love et al., 2014).

## Results

### Comparison of Young and Old Duplicate Genes in *Vernicia fordii*

According to the Ks values, we considered 463 and 465 duplicate gene pairs to be old duplicate genes (Ks: 1.3860–1.9935) and young duplicate genes (Ks: 0.0077–0.9489). Zhang et al. (2019) clarified that *Jatropha curcas* and *V. fordii* divergence occurred about 34.55 million years ago (Mya) (Ks = 0.52). These data suggested that young duplicate genes in *V. fordii* were formed before the divergence of *J. curcas* and *V. fordii*, and old duplicate genes in *V. fordii* were formed after the divergence of *J. curcas* and *V. fordii*.

**FIGURE 1:**
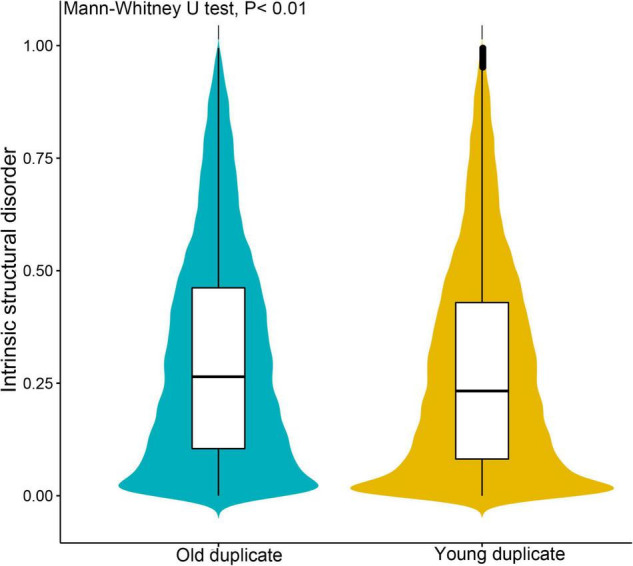
Comparison of intrinsic structural disorder (ISD) between young and old duplicate genes in *Vernicia fordii*. IUPred2A was used to estimate the ISD value with default parameters.

Subsequently, we investigated gene complexity, gene expression, and evolution patterns between young and old duplicate gene pairs, and found that these parameters differed between these genes. Young duplicate genes were expressed at higher levels in most tissues than old duplicate genes, and the gene expression breadth of young duplicate genes was also greater than old duplicate genes (Table 1). For codon usage bias, we found that there were similarities between young and old duplicate genes. The GC1 and GC3 content of young duplicate genes was found to be lower than that of old duplicate genes, and the polypeptide length of young duplicate genes was shorter than that of old duplicate genes. For ISD of proteins, the average ISD value of young duplicate genes was less than old duplicate genes (Figure 1). When Ka, Ks, and Ka/Ks were compared between young and old duplicate gene pairs, the values of Ka and Ks were higher for old duplicate gene pairs than for young duplicate gene pairs. However, the value of Ka/Ks for young duplicate gene pairs was higher than that of old duplicate gene pairs.

**TABLE 1:** Comparison of gene expression levels between old and young duplicate genes in *Vernicia fordii*.

	Young duplicate genes^a^	Old duplicate genes^a^	*P*-value
Stem	0.32992 ± 1.69659	1.29889 ± 19.01985	0.4732
Root	0.23075 ± 2.38534	0.26472 ± 3.32038	0.1732
Leaf	0.17276 ± 1.32728	0.54662 ± 5.19718	0.8877
10_WAF	0.16166 ± 0.93073	0.20305 ± 0.84959	0.641
15_WAF	0.61856 ± 9.47926	0.21273 ± 0.88024	0.7041
20_WAF	0.25948 ± 1.57819	0.22043 ± 1.60837	0.6372
25_WAF	0.21053 ± 2.19179	−0.03856 ± 5.21426	0.2906
30_WAF	−0.08646 ± 4.57126	0.03024 ± 8.43142	0.4939
C1	0.22886 ± 0.87381	0.09930 ± 0.82954	0.007285
C2	0.13126 ± 2.83309	0.18950 ± 1.12121	0.1539
C3	0.20570 ± 3.37712	0.17215 ± 2.26439	0.0423
C4	0.22757 ± 0.92811	−0.09541 ± 9.72672	0.1998
X1	0.39927 ± 2.21567	0.26412 ± 1.19283	0.05955
X2	0.22806 ± 1.96253	0.23484 ± 1.15092	0.09925
X3	0.22942 ± 4.14480	0.22514 ± 1.26084	0.7747
X4	0.24818 ± 1.52190	0.37369 ± 5.79896	0.1583
CX	0.40889 ± 2.65661	0.43914 ± 4.12942	0.137
Expression_ breadth	12.93952 ± 4.48514	12.91720 ± 4.56255	0.000173

*^a^Mean ± SD. 10_WAF, 15_WAF, 20_WAF, 25_WAF, and 30_WAF represent 10, 15, 20, and 25 weeks after flowering, respectively. C1, C2, C3, and C4 represent 30, 20, 10, 1 days before female flowering, respectively. X1, X2, X3, and X4 represent 30, 20, 10, 1 days before male flowering, respectively. CX means hermaphrodite.*

Previous studies have confirmed that there are different correlations among the gene complexity, expression pattern, and Ka and Ks between young and old duplicate genes (Song et al., 2019). To further understand this phenomenon in *V. fordii*, we performed a correlation analysis between young and old duplicate genes (Table 2 and Supplementary Figure 1). In young duplicate genes, there was no correlation between Ka, Ks, Ka/Ks, and the gene expression level of 17 different tissues. However, Ka and Ka/Ks have negatively correlated the gene expression level of 17 different tissues in old duplicate genes. We also noted that there are positive correlations among gene expression levels of 17 different tissues, and codon usage bias, gene expression breadth and GC3, and were negatively correlated GC2 in young duplicate genes. In old duplicate genes, the gene expression levels in 17 different tissues were positively correlated with gene-expression breadth, and were negatively correlated with GC2. However, codon usage bias, GC1, and GC3 were only positively correlated with the gene-expression level in partial tissues.

**TABLE 2:** Comparison of gene complexity and substitution rate between young and old duplicate genes in *Vernicia fordii*.

	Young duplicate genes*^a^*	Old duplicate genes*^a^*	*P*-value
Fop	0.36932 ± 0.03604	0.37620 ± 0.03576	0.003657
AA	380.13282 ± 262.40793	416.40538 ± 248.39721	0.0004001
GC1	49.19326 ± 4.69717	49.27901 ± 4.02039	0.8523
GC2	41.03073 ± 5.65408	40.07084 ± 5.02216	0.006975
GC3	36.50951 ± 5.79104	38.48259 ± 5.70046	2.06E-07
Ka	0.47117 ± 0.36448	0.38031 ± 0.26312	0.0003212
Ks	0.68886 ± 0.20766	1.60683 ± 0.16657	2.20E-16
Ka/Ks	0.84187 ± 1.05294	0.23830 ± 0.16605	2.20E-16

*^a^Mean ± SD; AA, polypeptide length; Fop, frequency of optimal codons; GC1/2/3, GC content at the first/second/third codon site; Ka/Ks, non-synonymous to synonymous substitution ratio; Ka, non-synonymous substitution rate per non-synonymous site; Ks, synonymous substitution rate per synonymous site.*

### Comparison of Gene Ontology Between Old and Young Duplicate Genes

Gene ontology terms are currently widely used by many researchers to understand the function and biological significance of genes (Botstein et al., 2000; Song et al., 2019). To gain insight into the potential functional divergence between young and old duplicate genes, we performed GO analyses of these genes in *V. fordii*. Compared to the old duplicate genes, the young duplicate genes contained more numbers of GO terms. We also noted that the GO types in young duplicate genes were more than that in old duplicate genes (Supplementary Figure 2 and Supplementary Tables 1–3). In the cellular component, young duplicate genes included more GO-specific terms associated with “membrane,” while old duplicate genes contained more GO-specific terms associated with “protein complex.” In the biological processes, young duplicate genes were more likely to mainly participate in “multi-organism cellular process,” but old duplicate genes were more mainly involved in “transport regulation” and “stress response.” In the molecular function, young duplicate genes preferentially carried out the “catalytic activity” function, while old duplicate genes are mainly involved in the “molecular adaptor” or “cyclin-dependent protein kinase activity” function (Supplementary Tables 1–3). Taken together, the analysis of GO terms suggests that young and old duplicate genes contain potential functional divergence during evolution.

### Location and Expression Divergence Analyses of Old and Young Duplicate Genes in *Vernicia fordii*

**FIGURE 2:**
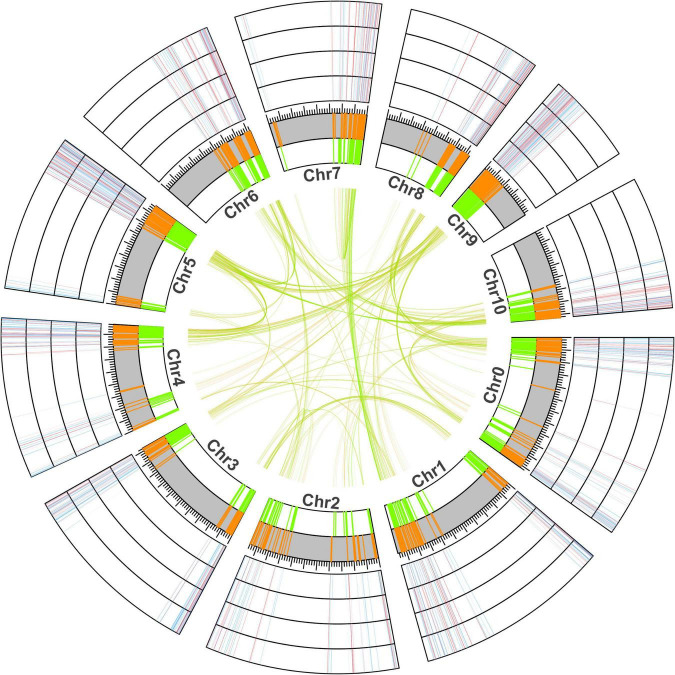
The location and expression analyses of young and old duplicate genes in *Vernicia fordii*. The green and orange represent the positional information of old and young duplicate genes on chromosomes in *V. fordii*, respectively. Blue and red represent the low and high expression levels of duplicate genes under *Fusarium* wilt disease, respectively. The outermost ring indicates F3, followed by F2, F1, and F0.

To further understand the chromosomal location of young and old duplicate genes, we obtained the GFF3 annotation file and performed location analysis in *V. fordii* (Figure 2). Our study suggested that young and old duplicate genes were mainly located in the end and beginning of chromosomes. In young and old duplicate genes, we found that 437 and 450 duplicate gene pairs were distributed among different chromosomes in *V. fordii*, respectively. Remarkably, a higher density of young and old duplicate genes was found on some chromosomes, such as chromosome 5 contained the highest number of duplicate genes (260), followed by chromosome 0 (214). In young duplicate genes, the highest number of old duplicate genes were mainly located in chromosome 5 (123) and chromosome 0 (106), respectively. However, old duplicate genes were located in chromosome 5 (137) and chromosome 9 (110), respectively.

**FIGURE 3:**
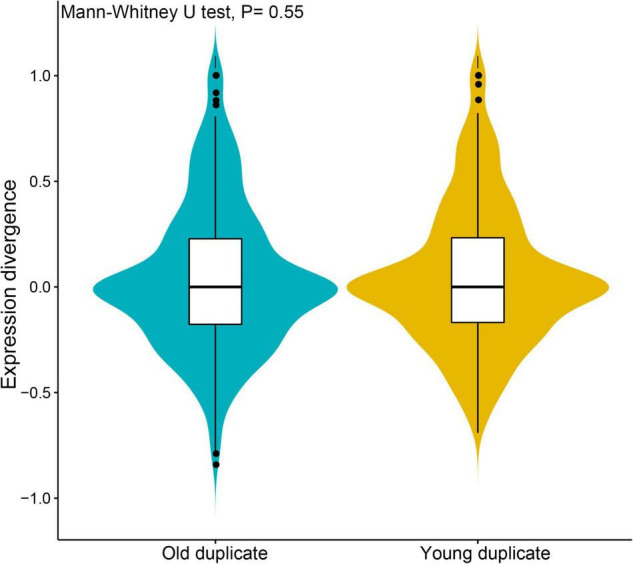
Expression divergence analyses of young and old duplicate genes during *Vernicia fordii* different tissues and/or development stages. The divergence and similarity between expression profiles of duplicate genes by using pearson’s correlation coefficient (r).

To further understand the degree of expression similarity between young and old duplicate genes, we analyzed the Pearson’s correlation coefficient (*r*) of each duplicate genes during *V. fordii* different tissues and/or development stages (Figure 3). The expression correlations for young duplicate genes demonstrated that the average value was *r* = 0.048, ranging from −0.069 to 1.000. By way of contrast, the correlations of old duplicate genes demonstrated a relatively low average value of 0.039 within a broad range of −0.119 to 1.000. However, the Mann–Whitney *U*-test found no significant difference between the two average *r* values (*p* = 0.55), which may be due to the relatively small number of samples. These data may further reflect some degree of ongoing functional divergence between duplicate genes during the long evolutionary period.

### Differential Gene Expression Between Young and Old Duplicate Genes Under *Fusarium* Wilt Disease

*Vernicia fordii* can produce biomass diesel, which is a promising industrial crop (Zhang et al., 2014; Liu et al., 2016). However, *Fusarium* wilt has caused devastating damage to *V. fordii* tress (Chen et al., 2016). To further understand the role of young and old duplicate genes in the resistance to *Fusarium* wilt, we performed a transcriptome analysis. A total of 72.79% (674/926) young and 82.80% (770/930) old genes expressed in the resistance to *Fusarium* wilt, indicating that these genes might play important roles in the resistance to *Fusarium* wilt (Figure 2). Compared to the young duplicate genes, the number of old duplicate genes was large, indicating that these genes involved in resistance to *Fusarium* wilt were not rapid expanded by duplication events during evolution.

**FIGURE 4:**
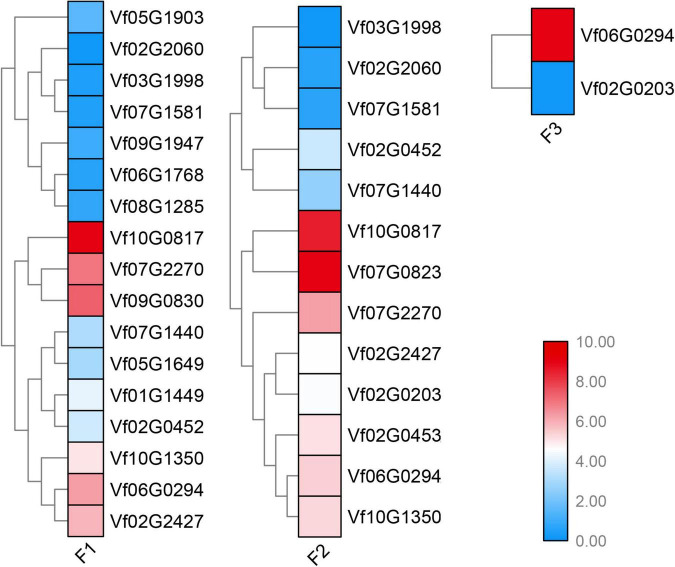
Young duplicate genes involved in response to *Fusarium* wilt disease stress. The same color font suggests that a duplicate gene was simultaneously involved in *Fusarium* wilt disease stress over more than one periods.

**FIGURE 5:**
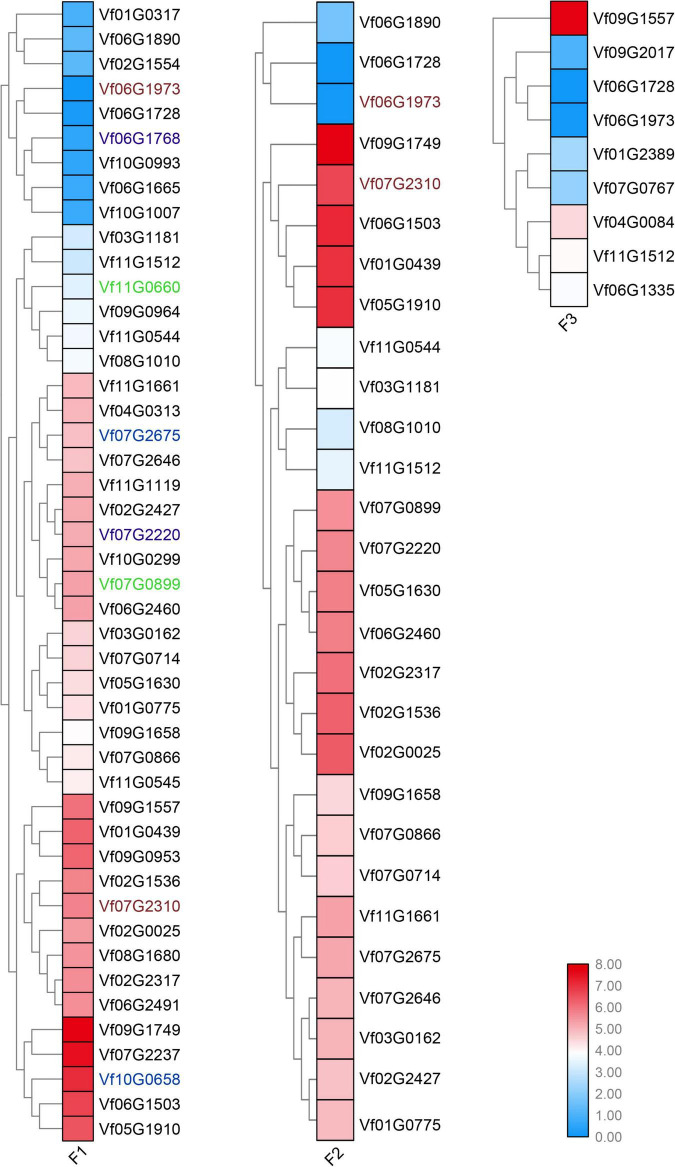
Old duplicate genes involved in response to *Fusarium* wilt disease stress. The same color font suggests that a duplicate gene was simultaneously involved in *Fusarium* wilt disease stress over more than one periods.

To determine the young and old genes with differential gene expression (DGE) during stages under *Fusarium* wilt disease, a significance threshold of 0.01 was applied using DESeq package. We revealed the differential expression modes between the young and old duplicate genes (Figures 4, 5). For old genes, we found that 25 and 58 genes were repressed and upregulated, respectively, at the early stage (F1 vs. F0), subsequent stage (F2 vs. F0), and finally stage (F3 vs. F0) after infection. However, for young genes, only 13 and 19 genes were repressed and induced, respectively, at the early stage (F1 vs. F0, F2 vs. F0, and F3 vs. F0) after infection. These results suggested that old genes might play a more important role in the resistance to *Fusarium* wilt than young genes. Remarkably, no young duplicate genes were observed in any three time periods. However, four old duplicate genes were detected at least one time period. Further, a divergent expression pattern was found in these old duplicate genes. The divergent expression patterns indicated that young and old duplicate genes contain different regulatory mechanisms in response to *Fusarium* wilt infection. Taken together, our study suggested that asymmetric function was found in young and old duplicate gene pairs under *Fusarium* wilt infection.

## Discussion

### Characteristics of the Young and Old Genes in *Vernicia fordii*

Surprisingly, there are few studies focused on young and old duplicate genes in plant genomes. Previous studies indicated that gene expression patterns are correlated with evolutionary patterns between young and old genes (Wolf et al., 2009; Cui et al., 2015; Gossmann et al., 2016). Unlike these studies, our analyses focused on gene complexity, expression profiles, functional divergence, and evolutionary patterns of young and old duplicate genes in *V. fordii*. In the present study, we found some interesting phenomena, including: (1) Young duplicate genes contained lower gene-expression levels than old duplicate genes; (2) Young duplicate genes possessed shorter polypeptide length than old duplicate genes; and (3) Young duplicate genes had relaxed purifying selection and lower ISD value than old duplicate genes. These data suggested that young and old duplicate genes differ not only in evolutionary patterns but also in expression profiles, selection pressure, and gene complexity, consistent with the results of articles published in animals, fungi and plants (Arendsee et al., 2014; Cui et al., 2015; Banerjee and Chakraborty, 2017; Wilson et al., 2017). For example, Song et al. (2019) found that there are no correlation between selective pressure and gene expression level, but selective pressure negatively correlated with the gene-expression level of old genes.

Previous studies have shown that the old genes were mainly influenced by natural selection, but young genes undergone multiple selection pressures (Vishnoi et al., 2010; Yin et al., 2016). Additionally, young genes were unstably expressed, while old genes were stably expressed and played essential functions in organisms (Chen et al., 2010, 2012; Hanada et al., 2018). Compared to the young genes, old genes have undergone strong purifying selection, which may help them maintain protein structure stability. In the present study, Ka and Ka/Ks of old duplicate genes negatively correlated with the gene expression, but no correlation was found between Ka, Ks, Ka/Ks, and the gene expression level in young duplicate genes. Remarkably, we found that the gene expression level of young duplicate genes was positively correlated with Fop, while was not correlated with Fop of old duplicate genes. These data suggested that although we used Ks values to classify young and old genes, most of the characteristics of these genes were consistent with previous studies, further confirming that the use of Ks values was a relatively reliable method for identification of young and old duplicate genes.

### Functional Analysis of the Young and Old Genes in *Vernicia fordii*

As a hemi-biotrophic root pathogen, *F. oxysporum* infects manly plants, such as *Musa nana*, *Solanum lycopersicum*, cotton, and *V. fordii* (Michielse and Rep, 2009). Tung wilt disease caused by *F. oxysporum* is considered to be the most deadly disease of *V. fordii*. To determine the potential function of young and old genes in the resistance to *Fusarium* wilt, a transcriptome analysis was performed during pathogen infection. In the young genes, most genes (72.79%, 674/926), especially transcription factors, are expressed in the resistance to *Fusarium* wilt. This phenomenon also exists in the old genes (82.80%, 770/930), which might indicate strong positive selection to maintain transcription factors. The comparative analysis revealed that the majority of duplicated genes, whether the young or old genes, presented similar expression patterns, and only a few duplicate genes presented divergent expression trends during pathogen infection. These data indicated that the most duplicate genes shared a similar function in resistance to pathogen infection, and only a few genes play the decisive roles by showing divergent expression trends.

Compared to young duplicate genes, the number of old duplicate genes was relatively small. However, the old duplicate genes were preferentially responded to biotic stress by GO terms results. The transcriptome analysis also suggested that old duplicate genes are involved in the response to *Fusarium* wilt. Previous studies showed that *V. fordii* has undergone only an ancient WGD, while not experienced a recent WGD event (Tang et al., 2016; Cao et al., 2019b). In the present study, old duplicate genes were mainly produced in an ancient whole genome duplication event. The climate of the earth has undergone tremendous changes in ancient times. The changing environment has increased biodiversity, including the number of parasites and microorganisms. In this case, plants adapt to parasites or pathogenic infections by increasing the resistance of the resistant biotic genes. In the present study, more old duplicate genes were authenticated during stages under *Fusarium* wilt disease. We propose that these genes that respond to biotic stress were increasingly produced in *V. fordii*, which was supported by the finding that old duplicate genes have participated in response to *Fusarium* wilt.

## Conclusion

In the present study, the properties of young and old duplicate genes were analyzed in *V. fordii* for the first time. Firstly, we generated a systematic investigation of young and old genes in *V. fordii*, which revealed common properties between our results and previous published papers. Next, we performed GO terms and examined the expression patterns of young and old duplicate genes in *V. fordii*, which suggested most young and old duplicate genes contained asymmetric function. These results will contribute to reveal the evolution and functional divergence of duplicate genes in *V. fordii*, and the identified important duplicate genes will provide key information to reveal targets for controlling wilt disease in *V. fordii*.

## Data Availability Statement

Publicly available datasets were analyzed in this study. Transcriptome data of tung tree (*Vernicia fordii*) can be found in NCBI with accession numbers of PRJNA483508, PRJNA445068, and GSE80228.

## Author Contributions

LJ, TF, XL, and JX designed and performed the experiments. LJ analyzed the data. LJ and JX wrote the manuscript. All authors reviewed and approved the final submission.

## Conflict of Interest

XL was employed by the company China Tobacco Hunan Industrial Co., Ltd. The remaining authors declare that the research was conducted in the absence of any commercial or financial relationships that could be construed as a potential conflict of interest. The handling editor declared a past co-authorship with one of the authors LJ.

## Publisher’s Note

All claims expressed in this article are solely those of the authors and do not necessarily represent those of their affiliated organizations, or those of the publisher, the editors and the reviewers. Any product that may be evaluated in this article, or claim that may be made by its manufacturer, is not guaranteed or endorsed by the publisher.

## Footnotes

1
http://codonw.sourceforge.net/

## Supplementary Material

Supplementary Figure 1 Correlation analysis of gene complexity, gene expression breadth, gene expression level, and substitution rate between young and old duplicate genes in *Vernicia fordii*. The figure was constructed using the gplots package in R. 10_WAF, 15_WAF, 20_WAF, 25_WAF, and 30_WAF represent 10, 15, 20, and 25 weeks after flowering, respectively. C1, C2, C3, and C4 represent 30, 20, 10, 1 days before female flowering, respectively. X1, X2, X3, and X4 represent 30, 20, 10, 1 days before male flowering, respectively. CX means hermaphrodite.

Supplementary Figure 2 Comparisons of the number of gene ontology (GO) terms between young and old duplicate genes in *Vernicia fordii*.

Supplementary Table 1 Specific gene ontology terms for cell components between young and old duplicate genes in *Vernicia fordii*.

Supplementary Table 2 Specific gene ontology terms for molecular functions between young and old duplicate genes in *Vernicia fordii*.

Supplementary Table 3 Specific gene ontology terms for biological processes between young and old duplicate genes in *Vernicia fordii*.
