# Supplementary figures and images for: Functional Heterogeneity of the Young and Old Duplicate Genes in Tung Tree (Vernicia fordii)
Source: Front Plant Sci. 2022 Jun 20;13:902649. doi: 10.3389/fpls.2022.902649 (PMC9253867; doi:10.3389/fpls.2022.902649)

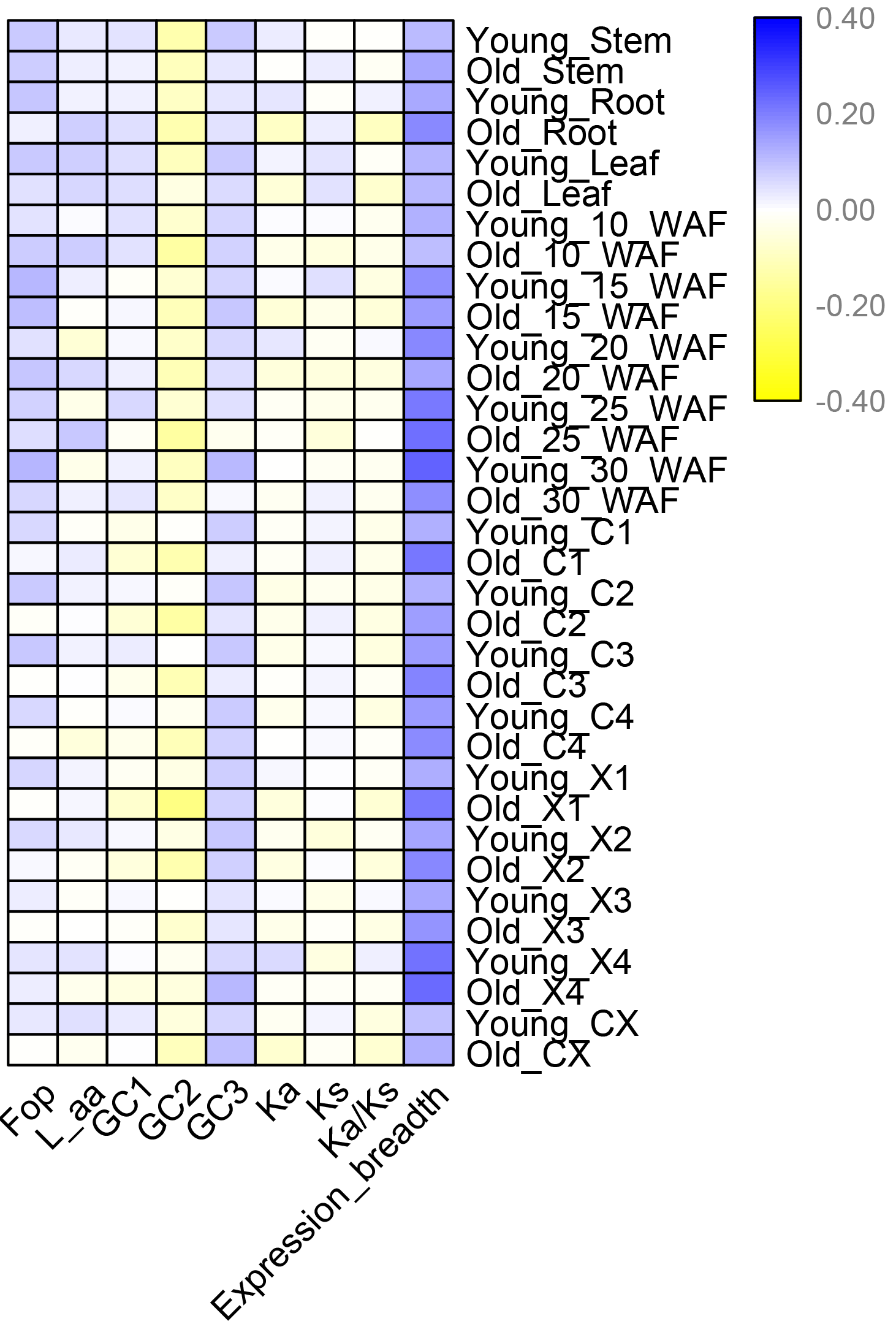

Supplement: Supplementary Figure 1 — Correlation analysis of gene complexity, gene expression breadth, gene expression level, and substitution rate between young and old duplicate genes in Vernicia fordii. The figure was constructed using the gplots package in R. 10_WAF, 15_WAF, 20_WAF, 25_WAF, and 30_WAF represent 10, 15, 20, and 25 weeks after flowering, respectively. C1, C2, C3, and C4 represent 30, 20, 10, 1 days before female flowering, respectively. X1, X2, X3, and X4 represent 30, 20, 10, 1 days before male flowering, respectively. CX means hermaphrodite. [file Image_1.TIF]

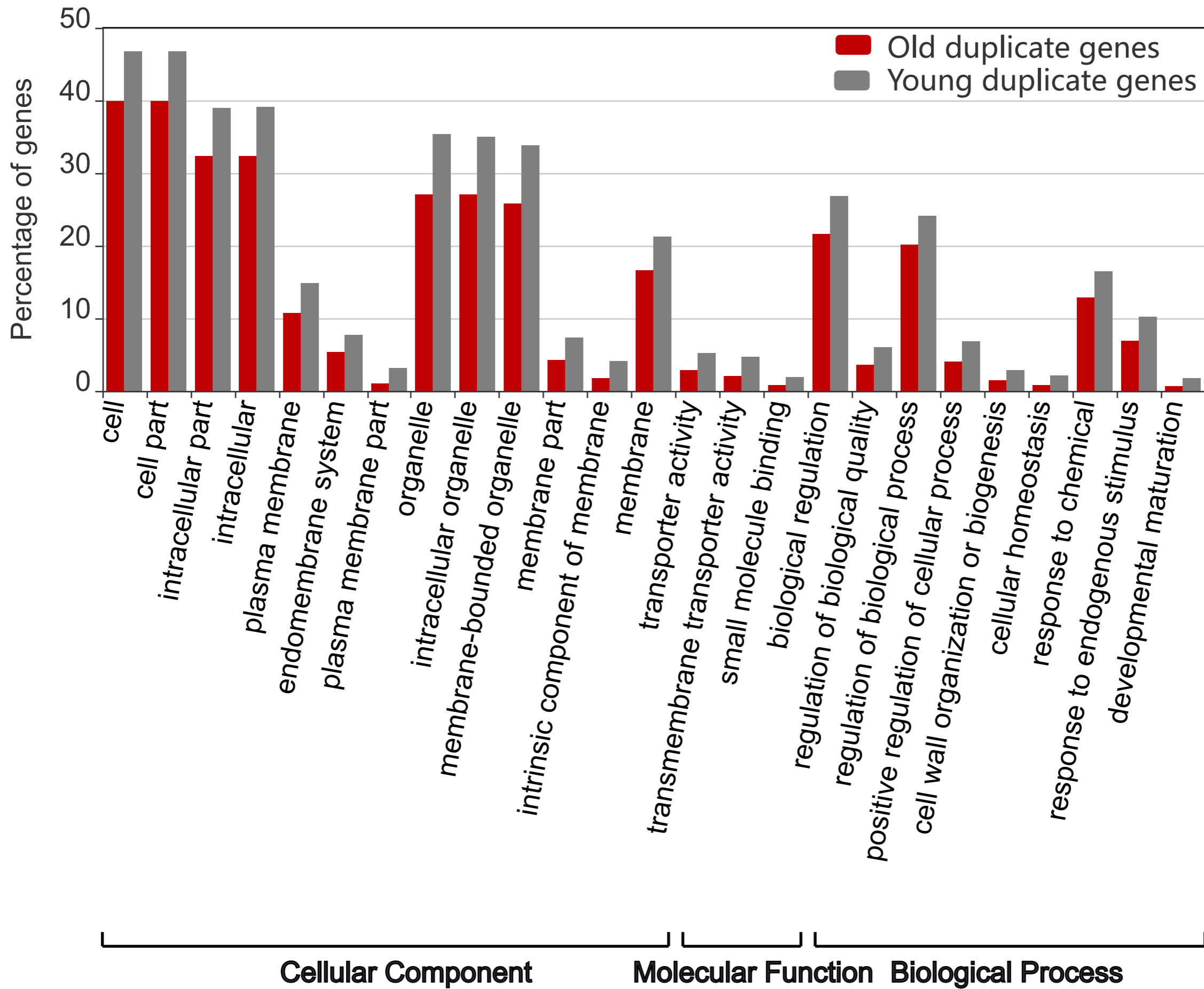

Supplement: Supplementary Figure 2 — Comparisons of the number of gene ontology (GO) terms between young and old duplicate genes in Vernicia fordii. [file Data_Sheet_1.PDF]
